# Effects of a Supraseasonal Drought on the Ecological Attributes of* Plagioscion squamosissimus* (Heckel, 1840) (Pisces, Sciaenidae) in a Brazilian Reservoir

**DOI:** 10.1155/2017/5930516

**Published:** 2017-02-23

**Authors:** Antonia E. F. Souza, Jônnata F. Oliveira, Danielle Peretti, Rodrigo Fernandes, Rodrigo S. Costa, José Luis Costa Novaes

**Affiliations:** ^1^Departamento de Ciências Animais, Laboratório de Ecologia de Peixes e Pesca Continental (UFERSA), BR 110, Km 47, Bairro Costa e Silva, 59625-900 Mossoró, RN, Brazil; ^2^Laboratório de Ictiologia, Universidade do Estado do Rio Grande do Norte (UERN), Av. Prof. Antônio Campos s/n, Bairro Costa e Silva, 59625-620 Mossoró, RN, Brazil; ^3^Departamento de Ciências Animais, Laboratório de Ecologia Quantitativa (UFERSA), BR 110, Km 47, Bairro Costa e Silva, 59625-900 Mossoró, RN, Brazil

## Abstract

The aim of this study was to evaluate the effect of a supraseasonal drought on the ecological attributes of* Plagioscion squamosissimus*. The fish were caught quarterly from February 2010 to November 2014 using gill nets in the reservoir of Santa Cruz, Rio Grande do Norte, Brazil. The abundance of the species was evaluated with the catch per unit effort (CPUE) metric and then correlated with the accumulated rainfall and water volume of the reservoir. The diet of the fish was evaluated using the feeding index (IAi). The proportional similarity index (PS_*i*_) was used to evaluate the variation in the niches of the fish. The body condition was inferred through the relative condition factor, and its variation was assessed with ANOVA. A reduction in the abundance of the species that were positively correlated with the reservoir water volume was observed. The diet of the fish comprised shrimp, gastropods, fish, insects, shrimp larvae, and vegetable matter, with shrimp being the major component. PS_*i*_ showed the occurrence of individual specialization during November 2013 and November 2014. The relative condition factor was not correlated with a reduction in the water volume of the reservoir. The supraseasonal drought did not affect the relative condition factor, diet, and the trophic niche, but it did affect the species abundance.

## 1. Introduction

Disturbances are often abrupt and sometimes random and are responsible for major damage to ecosystems, causing changes in the availability of resources and an impact on biological communities, such as the mortality of individuals [1, 2]. Disturbances can be of natural or anthropogenic origin, and their effects have repercussions from the individual to the ecosystem level [3]. Disturbances such as droughts are increasingly common in several regions of the world, particularly in arid and semiarid regions [4]. Among the effects of droughts on aquatic organisms are changes in the body condition of individuals, a decrease in the abundance and biomass of species, a decrease in the availability of resources, and intensification of competition and predation [5–7].

Directly or indirectly, disturbances influence the niches of species [8]. Despite the niche historically being considered a species or population attribute, it was noted that several species, rather than being categorized as generalists, in fact comprise individuals that only use the niche of a population subgroup [9]. One of the many factors influencing the intrapopulation variation of niches is individual specialization. Such specialization is found in a range of species, including both vertebrates and invertebrates [10]. An individual specialist is an individual that has adopted a narrower niche compared with the general population [10]. Among the factors driving niche creation is the variation in the diet among individuals, which highlights the importance of ecological interactions (e.g., intra- and interspecific competition, predation) to the development of niches [11].

The South American silver croaker,* Plagioscion squamosissimus* (Heckel, 1840), is a carnivorous Amazonian species of the Sciaenidae family (Owen, 1846) [12], whose diet consists primarily of fish and crustaceans [13]. The distribution of this species has been increasing because of its introduction into many Brazilian reservoirs, mainly to promote fisheries [14]. This species is one of the most abundant in semiarid reservoirs [15] and of the main fishery resources in the region, representing an important source of income and animal protein for human populations living close to the reservoirs [16].

The aim of this study was to evaluate the ecological responses of* P. squamosissimus* in a reservoir in the Brazilian semiarid region to drought conditions. The aspects analyzed were as follows: (1) the catch per unit effort (CPUE) in number and biomass; (2) the correlations among accumulated rainfall, reservoir water volume, and CPUE; (3) the occurrence of individual specialization; and (4) the relative condition factor. The hypotheses tested were as follows: (1) the species abundance and relative condition factor decrease with drought conditions and (2) the amplitude of the niche diversity is lower during the drought period.

## 2. Materials and Methods

### 2.1. Study Area

**Figure 1:**
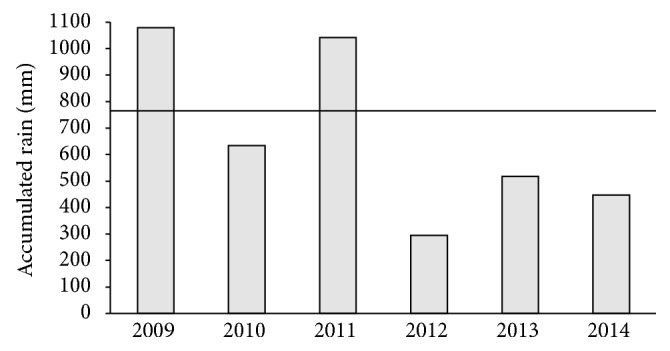
Variation in annual precipitation in the Santa Cruz of Apodi Reservoir, Brazil, between 2009 and 2014 [21].

**Figure 2:**
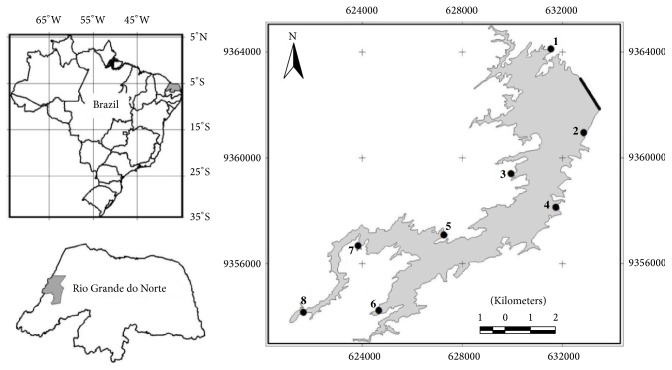
Location of the Santa Cruz of Apodi Reservoir in the hydrographic Apodi/Mossoró River basin, Brazil. The black line indicates the dam, and numbers (1–8) indicate the collection sites.

The hydrographic basin of the Oriental Northeast Atlantic occupies an area of 287,348 km^2^ and is characterized by caatinga vegetation and a semiarid climate; the basin has average annual rainfall of 750 mm, predominantly concentrated between February and May [17]. However, during the last 6 years, a decrease in the annual rainfall has been observed, with the exception of the years 2009 and 2011 (Figure 1). Among the small hydrographic basins located in the hydrographic basin of the Oriental Northeast Atlantic is the Apodi/Mossoró basin, with an area of 14,271 km^2^. The basin includes 20 important reservoirs, all with water volumes above 5 mi m^3^, constructed for human uses, including agriculture [18]. The Santa Cruz of Apodi Reservoir (05°45′45′′ S and 37°48′00′′  W; Figure 2), in the Apodi/Mossoró basin, is classified as oligomesotrophic and has an area of 341.3 ha and the capacity to accumulate up to 600 million m^3^ of water [19].

### 2.2. Data Collection

Data were collected quarterly between February 2010 and November 2014 at eight sampling points distributed along the reservoir (Figure 2). Fish were caught using gill nets placed at the reservoir bottom covering a total area of 301.8 m^2^; the nets had a mesh size of between 12 and 70 mm and were 15 m long and between 1.8 and 2.0 m high. At each point, the nets were set at 05:00 p.m. and removed at 05:00 a.m. the following day. For each individual collected, the total length (cm) (Lt) and weight (g) (Wt) were measured. The specimens were dissected, and only stomachs with food content were removed, fixed in 4% formaldehyde, and preserved in alcohol; the stomach contents were subsequently analyzed with a stereoscopic microscope. For the feeding study, only individuals caught between February 2011 and November 2014 were analyzed. The food items found were identified with the aid of specialized literature up to the lowest possible taxonomic level and then were grouped taxonomically for the analyses. The volume of the food items was measured by liquid displacement in a graduated cylinder [20]. The accumulated rainfall data (mm) and the accumulated water volume of the reservoir (in %) were used to infer the responses of* P. squamosissimus* to drought; data were obtained from the offices of the Agricultural Research Company of Rio Grande do Norte [21] in the city of Apodi and the Secretariat of the State of the Environment and Hydric Resources [17].

### 2.3. Data Analyses

To estimate the fish abundance, the numeric and biomass data of the fish captured were transformed into CPUE using the following formula: CPUE =* C*/*f*, where* C* is biomass (CPUEb) or* N* number of captured individuals (CPUEn) and *f* is effort in m^2^*∗*h, where m^2^ is 301.8 m^2^ and h is 12 h. The differences in the CPUE among months were tested with a one-way analysis of variance (ANOVA) followed by the Tukey test. The Pearson correlation coefficients between the water volume of the reservoir and accumulated rainfall with the CPUEb and CPUEn were calculated. For the study of the stomach contents, 382 specimens that presented food in the stomach were analyzed using the feeding index (IAi) [20], which combines the frequency of occurrence and volumetric methods [22, 23]. To evaluate whether the diet changed between months, the IAi values were first ordered by nonmetric multidimensional scaling (NMDS) [24] and then the differences between months were tested by permutational multivariate analysis of variance (PERMANOVA) [25]. The intrapopulation variation in trophic niche and individual specialization was quantified using the proportional similarity index (PS_*i*_), which calculates the degree of overlap between the diet of individuals and the population as a whole [9]. This metric is expressed by the value of each individual (PS_*i*_) and the average population (IS) [9]:

(1) $$\begin{array}{c} {\text{PS}}_{i}=1-0.5\underset{j}{\sum }\left|\;p_{ij}-q_{j}\;\right|=\underset{j}{\sum }\min\left(\;p_{ij},q_{i}\;\right), \end{array}$$

where *p*_*ij*_ is the frequency of prey type *j* in the diet of an individual and *q*_*j*_ is the frequency of prey type *j* in the diet of the population in general. If the amplitude of the IS reaches 1, the consumption of resources by individuals is equal to that of the population as a whole and there is an absence of specialization, whereas values close to 0 indicate strong specialization [26]. Values of probability (*P*) were obtained using the Monte Carlo procedure (999 simulations). The body condition was inferred by the relative condition factor calculated from the length-weight relationship variables. Body condition infers the changes in body weight based on the increase in length, with the relationship determined according to the following equation: *Wt* = *a* × *Lt*^*b*^, where *Wt* is the total weight (g), *Lt* is the total length (cm), *a* is the intercept, and *b* is the slope [27]. Differences in the relative condition factor among months were evaluated with a one-way ANOVA followed by a Tukey test; Pearson correlation coefficients were calculated to test the relationship between the relative condition factor and water volume of the reservoir. Statistical analyses were conducted using R software (Development Core Team, 20100) at a significance level of *P* < 0.05.

## 3. Results

**Figure 3:**
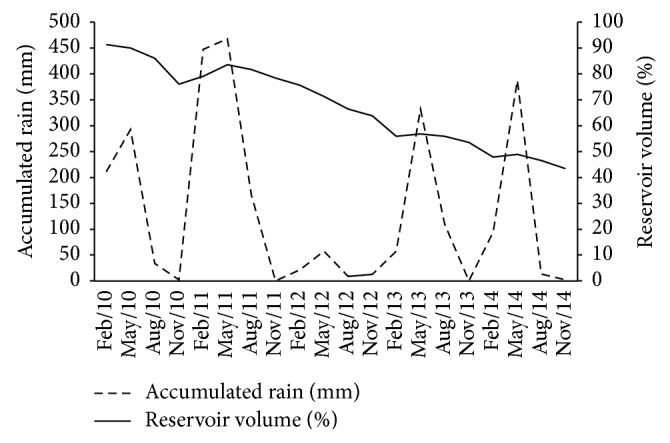
The quarterly variation in precipitation and water volume in the Santa Cruz of Apodi Reservoir, Brazil, between February 2010 and November 2014.

A considerable decrease in the water level was evident in the Santa Cruz of Apodi Reservoir during the study period, from 91.37% of its capacity in February 2010 to 43.40% of its capacity in November 2014. The precipitation ranged between 468 mm (May 2011) and 0 mm (November 2010, 2011, 2013, and 2014), with the highest amounts falling in February and May. The exception was in 2012, when precipitation was much lower than expected in February and May and the greatest reduction in water volume in the reservoir was recorded (Figure 3).

**Figure 4:**
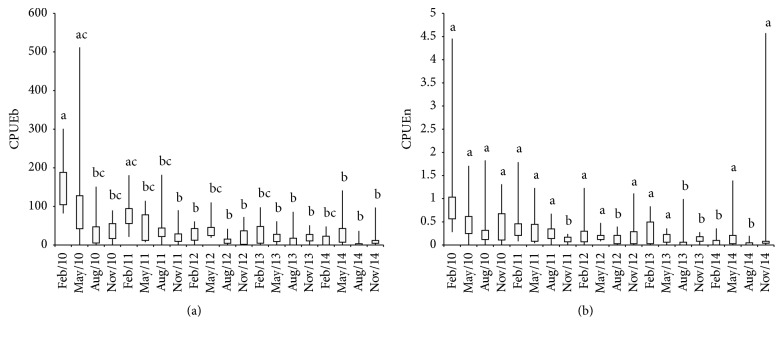
(a) Catch per unit effort estimated in biomass (CPUEb) and (b) number (CPUEn) of* Plagioscion squamosissimus* in the Santa Cruz of Apodi Reservoir, Brazil, between February 2010 and November 2014. The median is shown with a horizontal line inside the box. The minimum and maximum values are shown with short horizontal lines (“whiskers”). Different letters indicate statistical differences (Tukey, *P* < 0.05).

A total of 1,273 specimens were collected with a TL between 3.80 and 54.90 cm (average: 20.31; SD: 6.75) and a TW between 2.70 and 2,233.52 g (average: 120.31; SD: 141.95). The mean abundance in terms of CPUEb was 6,008.21 (g/301.8 m^2^*∗*12 h) and that in terms of CPUEn was 50.02 (ind./301.8 m^2^*∗*12 h). The highest monthly average CPUE (i.e., the highest estimated abundance) based on the highest biomass and highest number of captured individuals was in February 2010: CPUEb = 157.43 (g/301.8 m^2^*∗*12 h) and CPUEn = 1.18 (ind./301.8 m^2^*∗*12 h), respectively. The ANOVA was significant for CPUEb (*F* = 3.1; df = 51.42; *P* < 0.05) and CPUEn (*F* = 1.86; df = 51.29; *P* = 0.04) (Figure 4). The correlations between the reservoir water volume and CPUEb and CPUEn were positive and significant (*r* = 0.67; *P* = 0.01 and *r* = 0.50; *P* = 0.02, resp.). In contrast, there were no significant correlations between the mean annual precipitation and the mean CPUEb and CPUEn (*r* = 0.42; *P* = 0.06 and *r* = 0.29; *P* = 0.24, resp.).

**Figure 5:**
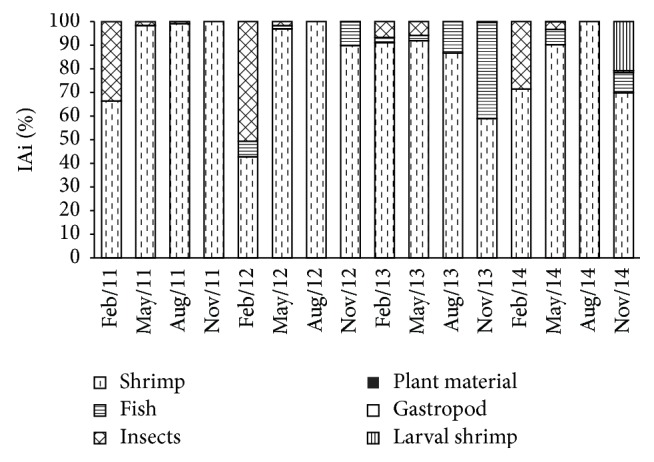
Quarterly percentages of the feeding index (IAi) for each food item category of the stomach contents of* Plagioscion squamosissimus* in the Santa Cruz of Apodi Reservoir, Brazil, between February 2011 and November 2014.

**Figure 6:**
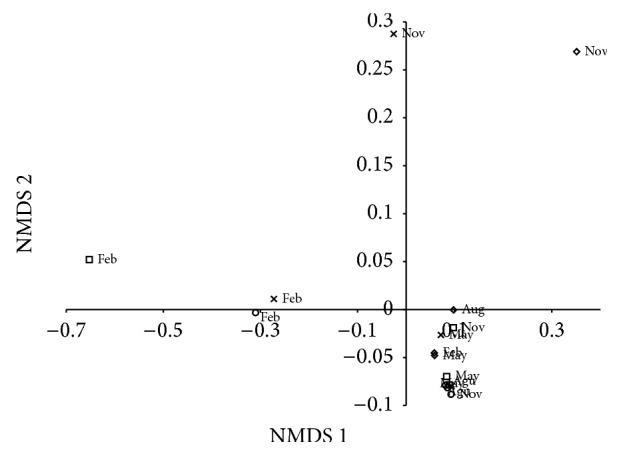
Nonmetric multidimensional scaling (NMDS – stress = 0.04; Axis 1 = 0.81 and Axis 2 = 0.29) of the monthly feeding index (IAi) of* Plagioscion squamosissimus* in the Santa Cruz of Apodi Reservoir, Rio Grande do Norte, Brazil, between February 2011 and November 2014. Feb: February; Aug: August; Nov: November; ○: 2011; □: 2012; ⋄: 2013; ×: 2014.

The diet of* P. squamosissimus* was composed of six items: (1) shrimp (*Macrobrachium amazonicum* Heller, 1862) (IAi = 84.6 (17.4), 42.76%–100%), (2) fish (scales, bones, and muscle) (IAi = 5.7 (10.2), 0%–40.72%), (3) insects (IAi = 8.3 (15.2), 0%–50.65%), (4) vegetable matter (IAi = 0.01 (0.03), 0%–0.36%), (5) gastropods (*Melanoides tuberculata* Müller, 1774) (IAi = 0.01 (0.04), 0%–0.21%), and (6) shrimp larvae (IAi = 1.3 (5.2), 0%–20.75%) (mean (standard deviation), minimum–maximum, resp.) (Figure 5). The NMDS ordination separated three groups: (i) months with high consumption of insects, specifically February 2011 (IAi = 33.62%), February 2012 (IAi = 50.65%), and February 2014 (IAi = 28.57%); (ii) months with high consumption of fish, specifically November 2013 (IAi = 40.72) and November 2014 (IAi = 8.54%); and (iii) other months with high consumption of shrimp (IAi < 69.82%) (Figure 6). Significant difference in IAi was found among months (*F* = 4.33; *P* = 0.01).

**Figure 7:**
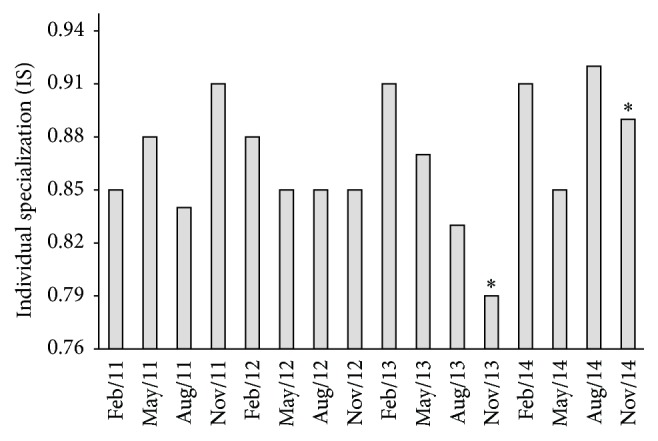
Quarterly values of the individual specialization index (IS) of the diet of* Plagioscion squamosissimus* samples in the Santa Cruz of Apodi Reservoir, Brazil, between February 2011 and November 2014. *∗* indicates *P* < 0.05.

The individual specialization index (IS) ranged from 0.79 to 0.92. The IS values were close to 1, indicating a high degree of similarity in the diets of individuals. In general, the population comprised generalists, except during November 2013 (IS = 0.79; *P* = 0.05) and November 2014 (IS = 0.89; *P* = 0.04), when the presence of specialists was recorded (Figure 7).

**Figure 8:**
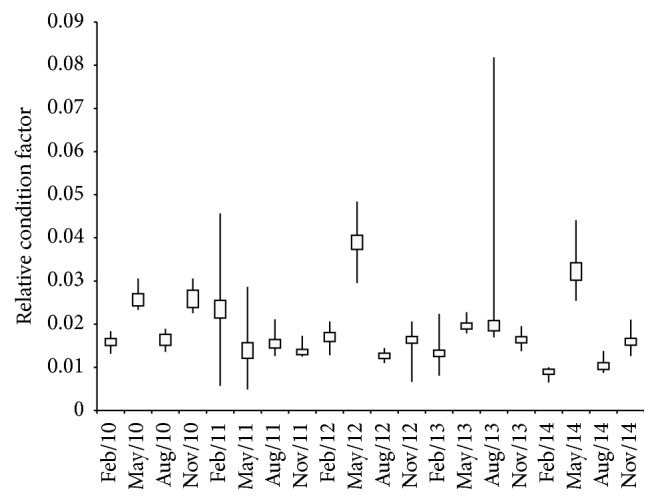
Variation in the relative condition factor of* Plagioscion squamosissimus* in the Santa Cruz of Apodi Reservoir, Rio Grande do Norte, Brazil, between February 2010 and November 2014. The median is shown with a horizontal line inside the box. The minimum and maximum values are shown with short horizontal lines (“whiskers”).

The relative condition factor ranged from 0.009 in February 2014 to 0.039 in May 2012 (Figure 8). The average relative condition factor also differed significantly among years (*F* = 278.9; df = 167.5; *P* < 0.05), and the Tukey test indicated differences among months (Table 1). There was no significant correlation between the water volume of the reservoir and relative condition factor (*r* = 0.13; *P* = 0.56).

## 4. Discussion

Although high precipitation occurred in 2009 and 2011, a large drop in the reservoir water volume occurred due to the rainfall scarcity from 2012 to 2015. The relationship of a reduction of the reservoir water volume with a decrease in the abundance of* P. squamosissimus* confirmed the hypothesis that the drought negatively affected the abundance of the species; these two variables were positively correlated. Similar results were described in the basin of the Guadalquivir River, Spain, where the water volume was the main environmental factor responsible for the reduction of the abundance of fish after an extended drought period [5]. A study of fish assemblages in streams in the Mira estuary, Portugal, similarly showed that the reduction in fish abundance was significantly influenced by water level [28]. As drought results in a reduction of the available habitat area, individuals of* P. squamosissimus* were possibly forced into a smaller volume of water, whereby a reduction in the availability of prey resulted in a shift from a generalist to a specialist diet. In agreement with this process, ecological interactions together with disturbances can cause a reduction in the abundance of species and may even result in permanent local effects [29]. Moreover, drought can impair the reproductive success and initial development of fish [7]. Therefore, directly and indirectly, disturbances such as drought can result in changes in patterns of species abundance [6], whereas recovery will depend on the life history characteristics of the affected species [30] as well as the frequency and intensity of the disturbance [31].

The diet composition analysis showed that* P. squamosissimus* had predominantly consumed shrimp (*Macrobrachium amazonicum* Heller, 1862), resulting in a considerable overlap in the NMDS ordination. Similar to the present study,* P. squamosissimus* was found to consume mainly shrimp in the Piató Lagoon, Rio Grande do Norte [32]. The consumption of shrimp has been reported for more than 50 species of Amazonian fish, including* P. squamosissimus* [33, 34]. It is likely that some life history aspects of* P. squamosissimus* in the selection of their food partly explain such results. With respect to the selection of the type and size of prey, several factors are involved, including the limitations imposed by the predator morphology, such as the size of the mouth, and behavioral strategies, such as those involved in the hunting and killing of prey [35].* Macrobrachium amazonicum* is an Amazonian species that can reach 30 cm in length and was introduced in northeastern reservoirs of Brazil [36, 37]. Its smaller body size compared with many other fish may be a factor affecting the food selection of* P. squamosissimus*. The relationship between the size of the prey and the predator has been noted as a key factor in determining the foraging success of aquatic communities [38]. An additional aspect that may have influenced the food selection of* P. squamosissimus* is the fact that when compared with fish (a main food item for the species in many aquatic environments), shrimp require little investment in terms of time and energy in seeking, capturing, killing, and consuming [39, 40]. The large consumption of shrimp may also be related to the fact that shrimp have lower mobility than fish, a characteristic that affects the chance of an encounter between predator and prey [41, 42]. Thus, considering that the investment in time and energy tends to increase when prey items are large, the consumption of small prey items such as shrimp may reflect efficient foraging [38, 43, 44].

During months when high rainfall was recorded (February 2011 and 2014), food items brought in by the rainfall runoff (e.g., insects) increased the contribution of less common items to the fish diet, as evidenced by ordination analysis. Similarly, the diversity of food resources increases during the rainy season, with insects being of central importance, which is attributed primarily to the rising water level and the succession of aquatic plants [45]. During the drier months, insects also contributed to the diet, as strong environmental limitations on their abundance were absent. Insects tolerate periods of water scarcity [46], including those of the Chironomidae and Culicidae families, which can tolerate anoxic conditions [47, 48]. The NMDS analysis showed that, during November 2013 and November 2014, the diet of* P. squamosissimus* also differed due to higher consumption of fish and shrimp larvae. The efficiency of a species' ability to seek, capture, and manipulate prey is related to the size of the prey; therefore, smaller prey are considered a highly important food resource of piscivorous species in the Upper Paraná River floodplain [49]. Thus, the supraseasonal drought did not affect the diet of* P. squamosissimus*. The differences observed in this study were related to cycle rain-dry of the region, even though it was weak during the studied period.

The high degree of similarity in the diets of individuals, mainly during November 2013 and November 2014, may have occurred due to the decrease in habitat area and the consequent ecological interactions such as intraspecific competition once individuals were forced into a restricted space. Ecological interactions can also promote changes in the niches of individuals [50]. Empirical and theoretical studies have shown that there is a positive association between intraspecific competition and individual specialization [11]. At the same time, although the premises postulated by optimal foraging theory assume that this interaction promotes an increase in the range of the niche for the population as whole as well as for individuals, many additional and yet unknown factors may restrict such an increase [51]. However, the present study did not find an association between drought and individual specialization, even though the reduction of the reservoir volume occurred over time and not only in a specific month. Moreover, in reservoirs, the feeding strategies of the predatory species are controlled to a larger degree by the availability and susceptibility of their prey than by their food preferences [52].

A decrease in the value of the fish condition factor is expected with a decrease in the water level that usually occurs during periods of drought [6, 53]; however, the relative condition factor of* P. squamosissimus* was not correlated with the reduction of the water volume of the reservoir. Previous research in the reservoir showed that a reduction of the water volume did not affect the fish condition factor because of an increase in the availability of food of better nutritional value [7]. In addition, the reduction of population abundance decreases competition for food, preventing the condition factor from decreasing.

## 5. Conclusion

The first hypothesis of the study was partially accepted; the abundance of* P. squamosissimus* decreased with the reduction of the water volume of the reservoir caused by the drought. However, no decrease in the relative condition factor was observed. The second hypothesis was not supported; the amplitude of the niche was not lower during the drought period.

## Figures and Tables

**Table 1:** Tukey test for differences in the relative condition factor between months for *Plagioscion squamosissimus *in the Santa Cruz of Apodi Reservoir, Brazil, between February 2010 and November 2014. Significant differences are in italics.

	Feb/10	May/10	Aug/10	Nov/10	Feb/11	May/11	Aug/11	Nov/11	Feb/12	May/12	Aug/12	Nov/12	Feb/13	May/13	Aug/13	Nov/13	Feb/14	May/14	Aug/14	Nov/14
Feb/10		*4.25E − 05*	1	*4.25E − 05*	*4.25E − 05*	0.8232	1	0.7805	0.9999	*4.25E − 05*	*0.04058*	1	0.3128	*0.006134*	*4.25E − 05*	1	*4.25E − 05*	*4.25E − 05*	4.35*E − 05*	1
May/10	15.71		*4.25E − 05*	1	0.544	*4.25E − 05*	*4.25E − 05*	*4.25E − 05*	*4.25E − 05*	*4.25E − 05*	*4.25E − 05*	*4.25E − 05*	*4.25E − 05*	*4.25E − 05*	*0.000694*	*4.25E − 05*	*4.25E − 05*	*4.25E − 05*	*4.25E − 05*	*4.25E − 05*
Aug/10	0.8754	14.84		*4.25E − 05*	*4.25E − 05*	0.3684	1	0.3207	1	*4.25E − 05*	*0.003871*	1	0.0609	0.05832	*4.31E − 05*	1	*4.25E − 05*	*4.25E − 05*	*4.25E − 05*	1
Nov/10	14.6	1.114	13.72		0.97	*4.25E − 05*	*4.25E − 05*	*4.25E − 05*	*4.25E − 05*	*4.25E − 05*	*4.25E − 05*	*4.25E − 05*	*4.25E − 05*	*4.26E − 05*	*0.01844*	*4.25E − 05*	*4.25E − 05*	*4.25E − 05*	*4.25E − 05*	*4.25E − 05*
Feb/11	12.1	3.607	11.23	2.493		*4.25E − 05*	*4.25E − 05*	*4.25E − 05*	*4.25E − 05*	*4.25E − 05*	*4.25E − 05*	*4.25E − 05*	*4.25E − 05*	*0.001511*	0.8733	*4.25E − 05*	*4.25E − 05*	*4.25E − 05*	*4.25E − 05*	*4.25E − 05*
May/11	3.058	18.77	3.934	17.66	15.16		0.9557	1	0.1032	*4.25E − 05*	0.9969	0.6263	1	*4.26E − 05*	*4.25E − 05*	0.4618	*4.84E − 05*	*4.25E − 05*	*0.0377*	0.8431
Aug/11	0.4659	16.18	1.341	15.06	12.57	2.592		0.9372	0.996	*4.25E − 05*	0.1128	1	0.557	*0.001507*	*4.25E − 05*	1	*4.25E − 05*	*4.25E − 05*	*4.96E − 05*	1
Nov/11	3.157	18.87	4.032	17.75	15.26	0.09856	2.691		0.08409	*4.25E − 05*	0.9984	0.5716	1	*4.26E − 05*	*4.25E − 05*	0.4092	*5.14E − 05*	*4.25E − 05*	*0.04758*	0.8028
Feb/12	1.62	14.09	0.7448	12.98	10.48	4.679	2.086	4.777		*4.25E − 05*	*0.000381*	1	*0.009352*	0.2482	*5.65E − 05*	1	*4.25E − 05*	*4.25E − 05*	*4.25E − 05*	0.9998
May/12	35.81	20.1	34.94	21.21	23.71	38.87	36.28	38.97	34.19		*4.25E − 05*	*4.25E − 05*	*4.25E − 05*	*4.25E − 05*	*4.25E − 05*	*4.25E − 05*	*4.25E − 05*	*4.25E − 05*	*4.25E − 05*	*4.25E − 05*
Aug/12	5.101	20.81	5.976	19.7	17.21	2.042	4.635	1.944	6.721	40.91		*0.01478*	1	*4.25E − 05*	*4.25E − 05*	*0.006526*	*0.007974*	*4.25E − 05*	0.8104	*0.04567*
Nov/12	0.4005	15.31	0.4749	14.2	11.7	3.459	0.8665	3.557	1.22	35.41	5.501		0.1609	*0.01835*	*4.26E − 05*	1	*4.25E − 05*	*4.25E − 05*	*4.27E − 05*	1
Feb/13	4.05	19.76	4.925	18.65	16.15	0.9912	3.584	0.8927	5.67	39.86	1.051	4.45		*4.25E − 05*	*4.25E − 05*	0.08968	*0.000319*	*4.25E − 05*	0.2726	0.3363
May/13	5.82	9.891	4.944	8.777	6.285	8.878	6.286	8.977	4.199	29.99	10.92	5.419	9.869		0.6797	*0.03853*	*4.25E − 05*	*4.25E − 05*	*4.25E − 05*	*0.005293*
Aug/13	9.18	6.531	8.304	5.417	2.924	12.24	9.646	12.34	7.56	26.63	14.28	8.779	13.23	3.36		*4.28E − 05*	*4.25E − 05*	*4.25E − 05*	*4.25E − 05*	*4.25E − 05*
Nov/13	0.697	15.01	0.1784	13.9	11.41	3.755	1.163	3.854	0.9232	35.11	5.798	0.2965	4.747	5.123	8.483		*4.25E − 05*	*4.25E − 05*	4.26*E − 05*	1
Feb/14	10.83	26.54	11.7	25.42	22.93	7.769	10.36	7.671	12.45	46.64	5.727	11.23	6.778	16.65	20.01	11.52		*4.25E − 05*	0.9478	*4.25E − 05*
May/14	25.42	9.71	24.55	10.82	13.32	28.48	25.89	28.58	23.8	10.39	30.52	25.02	29.47	19.6	16.24	24.72	36.25		*4.25E − 05*	*4.25E − 05*
Aug/14	8.19	23.9	9.065	22.79	20.29	5.132	7.724	5.033	9.81	44	3.089	8.591	4.14	14.01	17.37	8.887	2.638	33.61		*4.37E − 05*
Nov/14	0.05009	15.76	0.9255	14.65	12.15	3.008	0.4158	3.107	1.67	35.86	5.051	0.4506	4	5.87	9.23	0.7471	10.78	25.47	8.14	
